# Antioxidant activity and structural features of *Cinnamomum zeylanicum*

**DOI:** 10.1007/s13205-015-0296-3

**Published:** 2015-03-20

**Authors:** Tuhin Ghosh, Ankita Basu, Dipan Adhikari, Debnarayan Roy, Achintya Kumar Pal

**Affiliations:** 1 Department of Chemistry, Durgapur Government College, Durgapur, 713 214 West Bengal India; 2 Department of Chemistry, The University of Burdwan, Burdwan, 713 101 West Bengal India; 3 Department of Botany, Hooghly Mohsin College, Hooghly, Chinsurah, 712 101 West Bengal India; 4 Department of Zoology, Acharya Brojendra Nath Seal College, Cooch, Behar, 736 101 West Bengal India; 5 Department of Zoology, Durgapur Government College, Durgapur, 713 214 West Bengal India

**Keywords:** *Cinnamomum zeylanicum*, Bark, Polysaccharides, Size exclusion chromatography, Spectrometry, Antioxidant activity

## Abstract

The antioxidants in food materials have recently attracted researchers’ attention because many reports have shown that the oxidative stress is closely related to the aging process of the cells and acts as a trigger to various diseases including cancer. Since reactive oxygen species (ROS) is involved in initiating and promoting several diseases such as cancer and cardiovascular events, this study was designed to evaluate the antioxidant capacity of pectic polysaccharides extracted from the bark of *Cinnamomum zeylanicum,* locally known as Daruchini. An arabinogalactan (*A*), one partly methyl esterified galacturonic acid (*B*) and a neutral glucan (*C*) were isolated. The glucan is made up of β-(1 → 3)-linked glucopyranosyl residues and has a molecular mass of 7 kDa. The arabinogalactan is highly branched and has an average molecular mass of 40 kDa. The in vitro antioxidant capacity of the fractions was studied by ferric reducing antioxidant power (FRAP) and 1,1-diphenyl-2-picrylhydrazyl (DPPH) assays. The arabinogalactan (*A*) showed the highest potential followed by the uronic acid (*B*) and glucan (*C*). Taken together, these findings demonstrate that these polysaccharides could be used as natural antioxidants by the food industry.

**Electronic supplementary material:**

The online version of this article (doi:10.1007/s13205-015-0296-3) contains supplementary material, which is available to authorized users.

## Introduction

It is well known that although the raising levels of reactive oxygen species (ROS) and free radicals cause damage to nucleic acids, proteins, and membrane lipids, the antioxidants in diet would terminate attacks by the free radicals and reduce the risks of these diseases (Wiseman and Halliwell 1996). Reactive oxygen species are highly reactive molecules that are constantly produced by enzymatic reactions in cells. In normal physiological conditions, ROS are produced at low levels, which are necessary for maintaining normal cell functions, and the endogenous antioxidant defense systems of the body have the capacity to avert any harmful effects. However, free radicals-mediated modification of DNA, proteins, lipids, and small cellular molecules is associated with a number of pathological processes, including atherosclerosis, atherosclerosis, cancer, and rheumatoid arthritis (Halliwell and Gutteridge 1984). Therefore, antioxidants are important for bodily protection against oxidative stress. Lipid oxidation by reactive oxygen species (ROSs) such as super oxide anion, hydroxyl radicals, and hydrogen peroxide also causes a decrease in nutritional value of lipids, in their safety and appearance. In addition, it is the predominant cause of qualitative decay of foods, which leads to rancidity, toxicity, and destruction of biochemical components important in physiologic metabolism.

The different kinds of antioxidants were found in various levels in a variety of food materials including the spices and other ingredients used in preparation of food (Dreher and Junod 1996; Kinghorn and Compadre 2001; Oshima et al. 2003; Szabo and Ohshima 1996; Wang and Nixon 2001). *Cinnamomum zeylanicum* (family: Lauraceae), the perennial shrub of which is locally available in common market, known as Daruchini, grows predominantly in the northern parts of Bengal. All of the plant parts are aromatic, although only its leaves and barks are exploited commercially. The bark is widely used as spice and is an important Chinese medicine. For medicinal use, bark from mature trees grown in wild is preferred. For spice, the inner bark from young shoots as well as bark from older trees is used, which is peeled or scaped and dried. The antioxidants are considered as useful food additives in the food industry. Thus, the antioxidants would be used more widely as food additives to improve the quality of the cooked foods (Power et al. 2013). Although antioxidant activities have been reported for various food materials, most of the methods used to evaluate the activities gave only qualitative information, or relative value to positive control (Gülçin 2012; Halliwell 2013).

Recently, there is a considerable interest in the food industry and in the preventive medicine for the development of antioxidant from natural sources, such as marine flora and fauna, bacteria, fungi, and higher plants. Among them, higher plants represent one of the richest sources of bioactive compounds, and herbal derived products are increasingly used in medical and biochemical research (Mayer and Lehmann 2000). One particularly interesting feature of higher plants is their richness in pectic polysaccharides, the uses of which span from food, cosmetic, and pharmaceutical industries to microbiology and biotechnology (Renn 1997). These macromolecules have been proved to show a wide range of biological activities important to human health, for example, antiviral, antitumoral, antiinflammatory, and anticoagulant (Cumashi et al. 2007; Ghosh et al. 2009; Pomin and Mourão 2008). In recent years, several classes of polysaccharides have been demonstrated to show antioxidant activity too. The compounds tested included laminaran, alginic acid, fucoidan, and other unidentified macromolecules present in the extracts (Rocha de Souza et al. 2007; Ruperez et al. 2002; Wang et al. 2008).

In the present work, we describe the isolation and the structure elucidation of the pectic polysaccharides using spectroscopic and chemical methods and evaluation of in vitro antioxidant activity of arabinogalactan, glucan, and galacturonic acid present in *Cinnamomum zeylanicum*. These polysaccharides may represent a new approach for inhibiting the harm caused by excessive free radicals.

## Materials and methods

### Plant material and preliminary treatments

Barks of *Cinnamomum zeylanicum* (family: Lauraceae) were collected twice from the medicinal plant garden at Sunthelakhola, Jalpaiguri, West Bengal, India, in May, 2013, and April, 2014. The barks were washed thoroughly with tap water, dried by forced air circulation, and pulverized in a blender. The dried powder (200 g) was depigmented using sequential extraction with petroleum ether (24 h) and acetone (24 h) as solvent in a Soxhlet apparatus. The unextracted material was placed in a plastic beaker and air-dried to yield depigmented plant powder (DPP; 153 g).

### Extraction

Extraction of polysaccharide was conducted by stirring a suspension of this powder in water (pH 6.0) at 25–32 °C for 12 h. Separation of the residue from the extract was performed by filtration through glass filter (G-2). The residue was briefly washed with additional distilled water, and the wash was collected to maximize polysaccharide recovery. The insoluble material was extracted twice more under similar condition at a solute to solvent ratio of 1:100 (w/v). The combined liquid extract was dialyzed extensively against water and lyophilized. The recovered material was dissolved in water, diluted with ethanol (four volumes) and then collected by centrifugation (three times). The final pellet was dissolved in water and lyophilized to yield the water-extracted polysaccharide, named *A* (100 mg).

### Isolation of arabinogalactan protein (AGP) with β-glucosyl Yariv reagent

AGP was isolated according to Schultz et al. (2000) as described (Chatterjee et al. 2014). Briefly to a solution of *A* in 1 % NaCl (w/v) was added an equal volume of Yariv reagent also in 1 % NaCl. The mixture was kept at 4 °C for 18 h and then centrifuged. The pellet which was washed with 1 % NaCl followed by pure methanol (three times each) was then dried and treated with sodium metabisulfite (10 %). The resulting solution was dialyzed and freeze-dried to yield the arabinogalactan proteins (AGPs).

### Size exclusion chromatography (SEC)

The water-extracted fraction *A* and the acid extracted fraction *B* were chromatographed on a Sephacryl S-100 column (2.6 × 90 cm; Amersham Biosciences AB, Uppsala, Sweden) using 0.5 M sodium acetate buffer (pH 5.5) as eluent. The flow rate of the column was 0.5 ml min^−1^, and fractions of 10 ml were collected and checked by the phenol–sulfuric acid reaction (Dubois et al. 1956). Elution of polysaccharide was expressed as a function of the partition coefficient *K*_av_ [*K*_av_ = (*V*_e_ − V_0_)/(*V*_t_ − *V*_0_) with *V*_t_ and *V*_0_ being the total and void volume of the column determined as the elution volume of potassium hydrogen phthalate and dextran, respectively, and *V*_e_ is the elution volume of the sample].

### Chemical analysis

Chemicals used were analytical grade or best available. All experiments were conducted at least in duplicate, and the mean and standard deviation were directly calculated from the functions present in excel program. Evaporations were performed under diminished pressure at ~45 °C (bath), and small volume of aqueous solutions was lyophilized. Dialysis against distilled water was performed with continuous stirring, toluene being added to inhibit microbial growth. Moisture was determined by drying ground material in an air-circulated oven at 110 °C for 3 h. Gas liquid chromatography (GLC) was carried out on a Shimadzu GC-17A (Shimadzu, Kyoto, Japan) gas chromatograph equipped with FID, and nitrogen was used as a carrier gas. Gas liquid chromatography–mass spectrometry (GLC–MS) analysis was carried out on a Shimadzu QP 5050 A, Shimadzu, and the ionization potential was 70 eV.

### Sugar analysis

Total sugars and uronic acids were determined by the phenol–sulfuric acid (Dubois et al. 1956) and m-hydroxydiphenyl (Ahmed and Labavitch 1977) assay, respectively. All fractions were hydrolyzed with 2 M trifluoro acetic acid (2 h, 100 °C) for measurement of individual neutral sugar. Sugars were reduced, acetylated, and analyzed as their alditol acetate by GLC (Blakeney et al. 1983) on columns of 3 % SP-2340 on Supelcoport 100–120 mesh, and DB-225 (JW) and by GLC-MS. Myo-inositol was used as an internal standard. Sugars in the acid hydrolysate were also analyzed by thin layer chromatography as described (Ray et al. 2013). DPP was hydrolyzed with 2 M trifluoro acetic acid (2 h at 100 °C) for soluble substances. For insoluble residues, this hydrolysis was followed by a treatment of the residue with 72 % (w/w) H_2_SO_4_ for 1 h at room temperature and then with 1 M H_2_SO_4_ for 2 h at 100 °C. Alternatively, trimethylsilyl (TMS) derivatives of methyl glycosides were analyzed by gas chromatography.

### Amino acid analysis

Protein content was measured in the insoluble residue by estimating total nitrogen (Kjeldhal method) and multiplying the value with 6.25. In the soluble material, the Bradford method was assayed using bovine serum albumin as standard. Amino acids were released by hydrolysis with 6 M HCl at 110 °C for 22 h in a sealed tube. Protections were done for cysteine, methionine, and tyrosine using proper protecting reagents. The liberated amino acids were analyzed by Hitachi’s Model L-8900 Amino Acid analyzer.

### Linkage analysis

The polymer AF2 (5 mg) was subjected to three rounds of methylation (Blakeney and Stone 1985). Permethylated samples were hydrolyzed, converted into their partially methylated alditol acetates, and analyzed by GLC and GLC–MS as described (Ray et al. 2013).

### Spectroscopy

Recording of IR spectra was carried out as described previously (Ray et al. 2013) and recorded on a Fourier transform IR spectrophotometer (FT-IR spectrometer, Spectrum RX 1, PerkinElmer, USA). The ^1^H NMR spectra were recorded on a Bruker 600 spectrometer (Bruker Biospin AG, Fallanden, Switzerland) operating at 600 MHz, respectively, for ^1^H. The samples (~10 mg of each) were heated (at 80 °C for 30 min) with water (1 mL) and centrifuged, and the resulting supernatant was lyophilized. The freeze-dried sample was deuterium-exchanged by lyophilization with D_2_O (Sigma–Aldrich) and then examined in D_2_O (99.96 atom% D).

### Ferric ion reducing/antioxidant power (FRAP) assay

The FRAP assay was conducted according to Benzie and Strain (1996) as modified by Pulido et al. (2000). Briefly, the oxidant was prepared by mixing 2.5 mL of a 10 mM TPTZ [2,4,6-tri(2-pyridyl-5-triazine) Fluka Chemicals, Madrid, Spain] solution in 40 mM HCl with 25 mL of 0.3 M acetate buffer (pH 3.6) and 2.5 mL of 20 mM·FeCl_3_·6H_2_O. The final solution has Fe(III) of 1.67 mM and TPTZ of 0.83 mM. To measure FRAP value, 900 μL of freshly prepared FRAP reagent was warmed to 37 °C and a reagent blank reading was taken at 593 nm; then, 30 μL of test sample and 90 μL of distilled water were added. Absorbance readings were taken after 0.5 s and every 15 s until 30 min using a Shimadzu UV-1601 (PC) Spectrophotometer. The change in absorbance (∆*A* = *A*_30min_ − A_0min_) was calculated and related to Δ*A* of an Fe(II) standard solution. Aqueous solutions of known Fe(II) concentrations (100–2000 μM/L FeSO_4_·7H_2_O) were used for calibration.

### Scavenging capability for 1,1-Diphenyl-2-picrylhydrazyl (DPPH) Radicals

The method reported by Shimada et al. (1992) was adopted for measurement of free radical scavenging capability. To each 4 mL of sample solution, 1 mL of freshly prepared methanolic DMSO solution of DPPH (0.5 mM) was added, mixed well, and then let stand for 30 min at room temperature in the dark. The absorbance of the resulting was recorded at 517 nm. Butylated hydroxyanisole (BHA) was used as reference compound. The capability to scavenge the DPPH radical was calculated using the following equation:

$$ {\text{Scavenging effect (}}\% )\; = \left[1 - \frac{\text{absorbance of sample at 517 nm}}{\text{absorbance of control at 517 nm}}\right] \times 100 $$

### Statistical analysis

Origin 6 software (Microcal Software Inc., USA) was used for data analysis. All experiments were repeated three times, and data were presented as mean ± SD for three replications for each sample. The Fisher least significance test was used to check the equality of variances and one-way ANOVA was used to estimate the statistically significant difference (*p* ≤ 0.05).

## Results and discussion

### Chemical characterization of the pectic arabinogalactan

#### Isolation and chemical composition

**Fig. 1:**
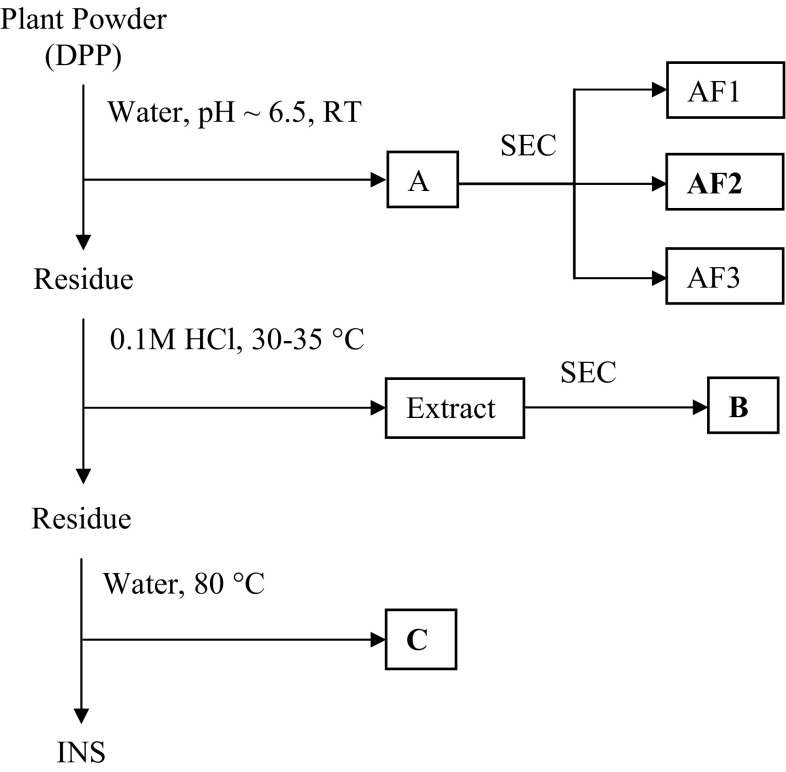
Scheme for the extraction and purification of polysaccharides from the barks of *Cinnamomum zeylanicum*

The main goal of this research was to analyze the water-extracted polysaccharide generated from the barks of *Cinnamomum zeylanicum* and to study its antioxidant activity (Fig. 1). In Indian Ayurvedic system of medicine, a decoction of its bark in water is used as herbal remedy for chronic asthma, bronchitis, cardiac disorder, and fevers (Mandal et al. 2007); therefore, barks of this herb was extracted with water. The yield of the water-extracted polymer (named as *A*), after fractional precipitation with ethanol, was 40 mg per gram of bark. The use of cold water, in principle, may exclude the extraction of physiologically inactive starch, present in the leaves. Sugar composition analysis revealed that fraction *A* consisted mainly of arabinose and galactose as the major neutral sugar together with smaller amount of glucose, rhamnose, mannose, and xylose units (Table 1). The uronide content of the fraction B is 7.5 % (w/w). Thin layer chromatographic analysis of the monosaccharides present in the hydrolysate indicates the presence of an uronic acid with *R*_f_ values similar to that of galacturonic acid. GLC analysis of the TMS derivatives of the derived methyl glycosides confirmed this result, but it also shows the presence of traces of glucuronic acid.

**Table 1:** Yield and molar sugar composition of *Cinnamomum zeylanicum* bark and of fractions obtained there from

	*A*	AGP	AF1	AF2	AF3	*B*	*C*
Yield^a^	25	5	2.5	5	2	3.5	2
Total Sugar^b^	62	51	87	72	68	49	57
Uronic Acid^b^	7	tr	5	25	2	15	13
Rhamnose^c^	2	1	3	2	2	1	7
Arabinose^c^	27	34	28	30	30	32	6
Xylose^c^	1	tr	1	1	tr	tr	tr
Mannose^c^	1	1	1	tr	tr	tr	tr
Galactose^c^	62	64	61	62	61	62	1
Glucose^c^	7	tr	6	5	7	5	86
GlcA^c^	48	tr	tr	42	tr	63	65
GalA^c^	52	tr	tr	58	tr	37	35

Water-extracted polysaccharide-containing fraction (*A*) isolated from *Cinnamomum zeylanicum* barks, the arabinogalactan protein (AGP) and of fractions (AF1, AF3), *B* and *C* derived therefrom by size exclusion chromatography

*tr* trace

^b^Weight percentage of dry weight

^c^Percentage weight of fraction dry weight

^d^Molar percentage of neutral sugars

Considering that the water-extracted polymeric fraction (*A*) contained galactosyl and arabinosyl residues as the major sugars, and its protein content is 24 % (w/w), we have tested its reactivity with *β*-glucosyl Yariv reagent. We found that a part of A was precipitable with the Yariv reagent. Sugar compositional analysis of this precipitate (named AGPs) shows that it consisted mainly of galactose residues and, to a lesser extent, arabinose residues, confirming the presence of AGP (Table 1). This material also contained mannose residues probably originating from *N*-glycans. The amino acid composition of proteins associated with fraction *A* showed that glutamic acid/glutamine (37.5 %), alanine (14.3 %), serine (9.5 %), and glycine (6.4 %) were the major constituents along with trace amount of proline and hydroxyproline.

#### Size exclusion chromatography (SEC)

**Fig. 2:**
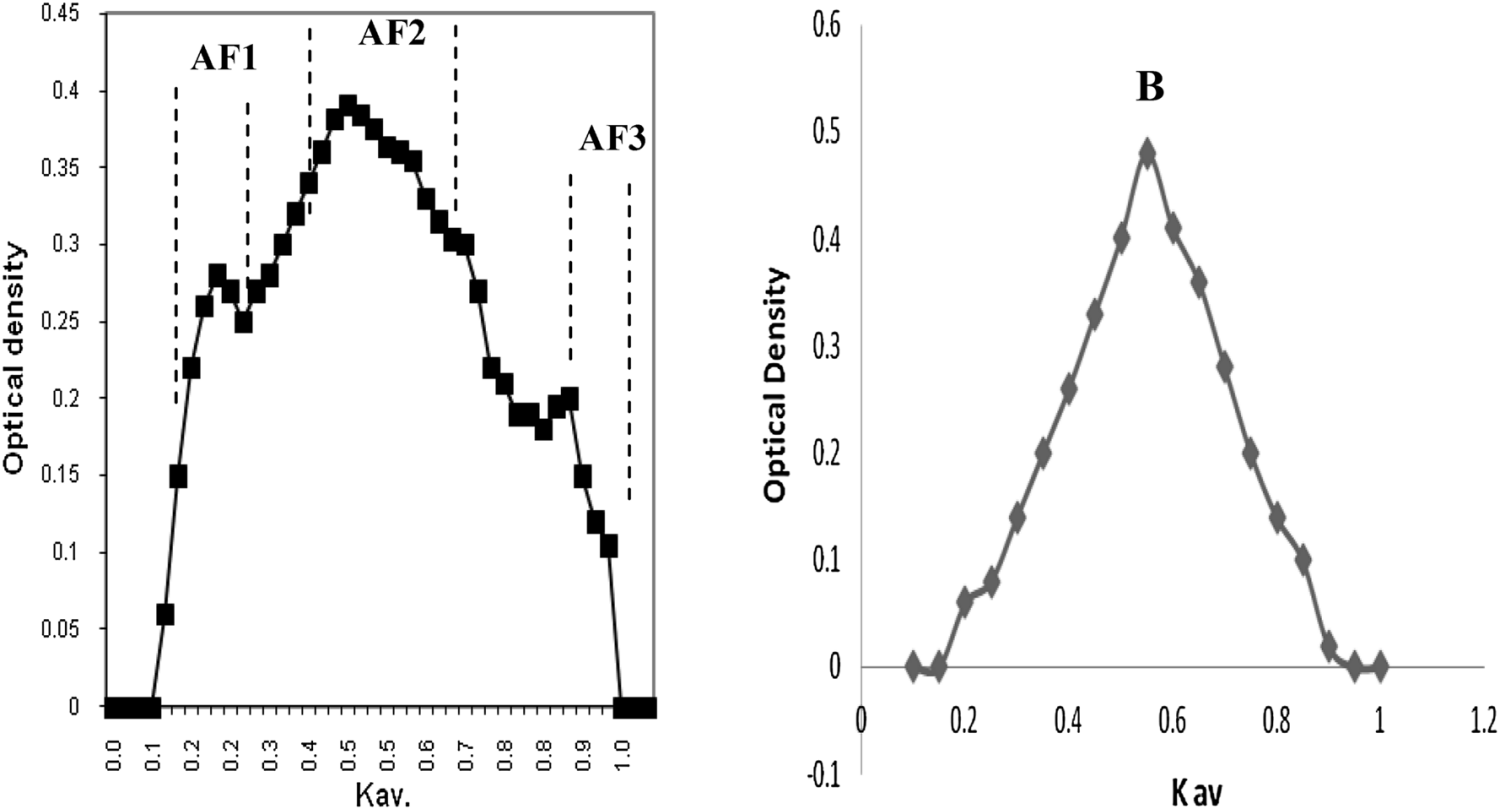
Elution profile of the water-extracted polysaccharide obtained from *Cinnamomum zeylanicum* barks on Sephacryl S-100 column with 500 mM sodium acetate buffer (pH 5.5) at 30 ml/h. Collected fractions were analyzed for total sugar content by phenol–sulfuric acid. Elution of polysaccharide was expressed as a function of the partition coefficient *K*_av_ [*K*_av_ = (*V*_e_ − *V*_o_)/(*V*_t_ − *V*_o_) with *V*_t_ and *V*_o_ being the total and void volume of the column determined as the elution volume of potassium hydrogen phthalate and dextran (500 kDa), respectively, and *V*_e_ is the elution volume of the sample]

Fraction *A* was subjected to further chemical analysis. First, this fraction was submitted to SEC onto Sephacryl S-100 (Fig. 2), which yielded three overlapping subfractions (F1, F2, and F3). All the subfractions had similar monosaccharide compositions (Table 1). F1, F2, and F3 represented 16, 72, and 12 %, respectively, of the total sugar recovered from the column. They also had similar ^1^H NMR spectra. The only difference between these three samples, as judged by size exclusion chromatography, seems to be the molecular weight. The fractionation range of the Sephacryl S-100 column was 1000–300,000 Da for globular protein and about the same for dextrans.

#### Linkage analysis

Methylation analysis of the polysaccharide from *Cinnamomum zeylanicum* yielded a variety of partially methylated alditol acetates (Table 2). The results suggest that galactopyranosyl residues are 1,3- and 1,3,6-linked, whereas arabinofuranosyl units are 1,5- and 1,3,5-linked. The presence of 1,2- and 1,2,4-linked rhamnopyranosyl residues were also indicated. This result suggests the presence of pectic arabinogalactan. This fraction also contained 1,4-linked glucopyranosyl residues (Supplementary Fig. 1).

**Table 2:** Partially methylated alditol acetates derived from the pectic arabinogalactan (AF2) of *Cinnamomum zeylanicum*

Linkages^a^	m/z	Peak area^b^
T-Ara*f*	43, 45, 102, 118, 129, 161	5
(1 → 2)-Ara*f*	43, 45, 101, 130, 161, 190	1
(1 → 5)-Ara*f*	43, 102, 118, 129, 162, 189, 233	30
(1 → 3,5)-Ara*f*	43, 118, 201, 261	18
T-Rha*p*	43, 102, 118, 131, 162, 175	2
(1 → 2)-Rha*p*	43, 130, 131, 174, 175, 190, 234	2
(1 → 3)-Rha*p*	43, 118, 131, 174, 234	2
(1 → 2,4)-Rha*p*	43, 130, 143, 190, 203	2
(1 → 4)-Glc*p*	43, 45, 102, 113, 118, 130, 162, 233	8
T-Gal*p*	43, 45, 101, 102, 118, 129, 145, 161, 162, 205	Tr^c^
(1 → 3)-Gal*p*	43, 45, 101, 118, 129, 161, 174, 234	13
(1 → 6)-Gal*p*	43, 102, 118, 129, 162, 189, 233	2
(1 → 3,6)-Gal*p*	43, 118, 174, 189, 234	15

^a^Linkage of monosaccharides. T-Ara*f* denotes 1,4-di-*O*-acetyl-2,3,5-tri-*O*-methylarabinitol, etc

^b^Percentage of total area of the identified peaks

^c^ *Tr* trace

#### Fourier transform infrared spectroscopy (FT–IR)

**Fig. 3:**
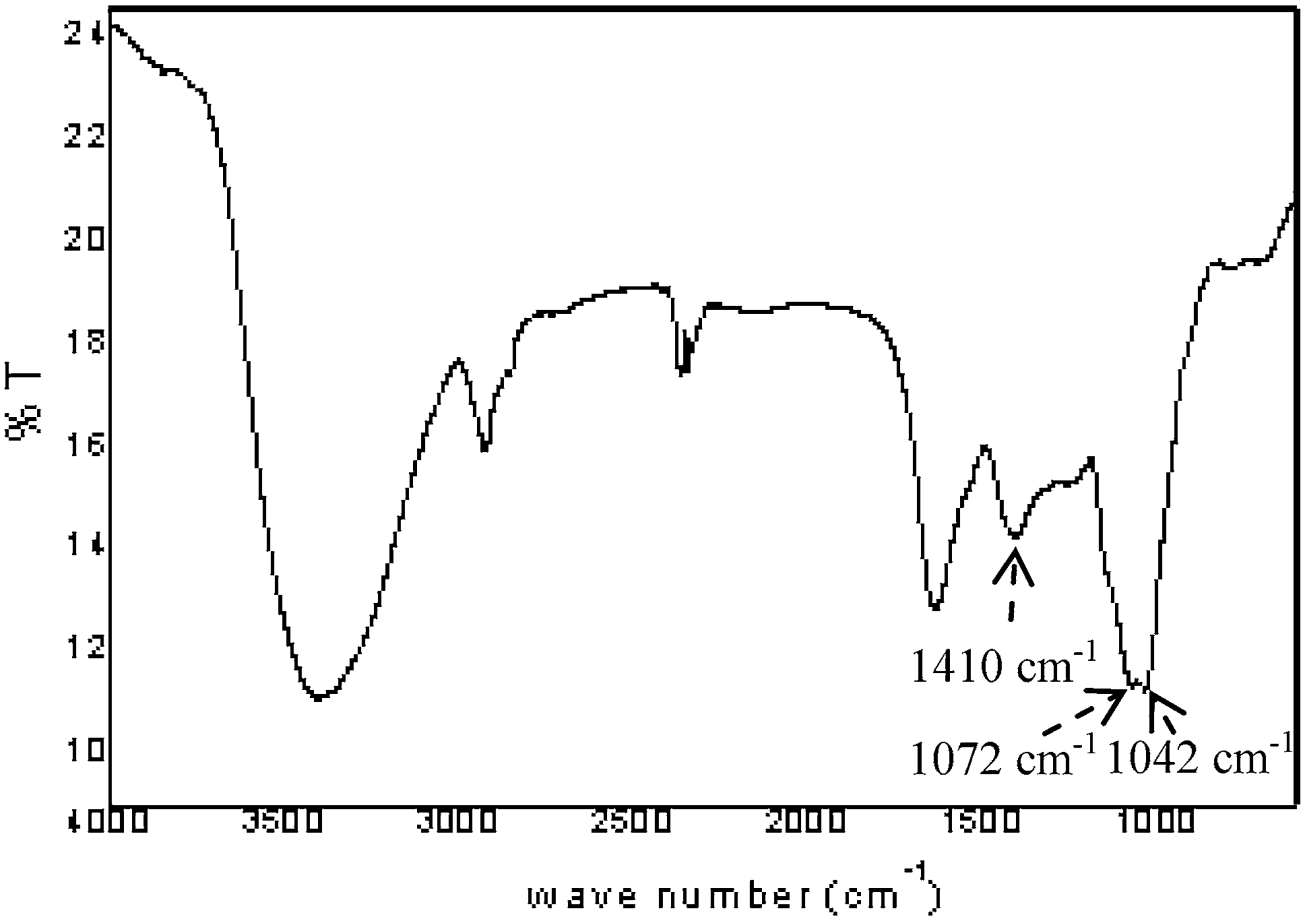
FT–IR spectrum of the pectic arabinogalactan (AF2) isolated from *Cinnamomum zeylanicum*

FT–IR spectroscopy is a valuable tool for determining the bulk structural features of polysaccharide (Kacurakova et al. 2000). The FT–IR spectrum of A fraction shows (1) a broad band between 3600 and 3000 cm^−1^ corresponding to vibrations of the hydroxylic groups, (2) methyl and methylene group vibrations around 2930 cm^−1^, and (3) a band in the region 1410 cm^−1^ related to the carbonyl stretching of the carboxylate anion (Fig. 3). Structural features arising from particular conformations around the glycosidic bonds of the polysaccharide were observable in the 850–1200 cm^−1^ region (Kacurakova et al. 2000). The absorbance at 1042 cm^−1^ and a band at 1072 cm^−1^ corresponding to arabinosyl and galactosyl were also present in the spectrum (Coimbra et al. 1998).

#### ^1^H NMR spectroscopy

**Fig. 4:**
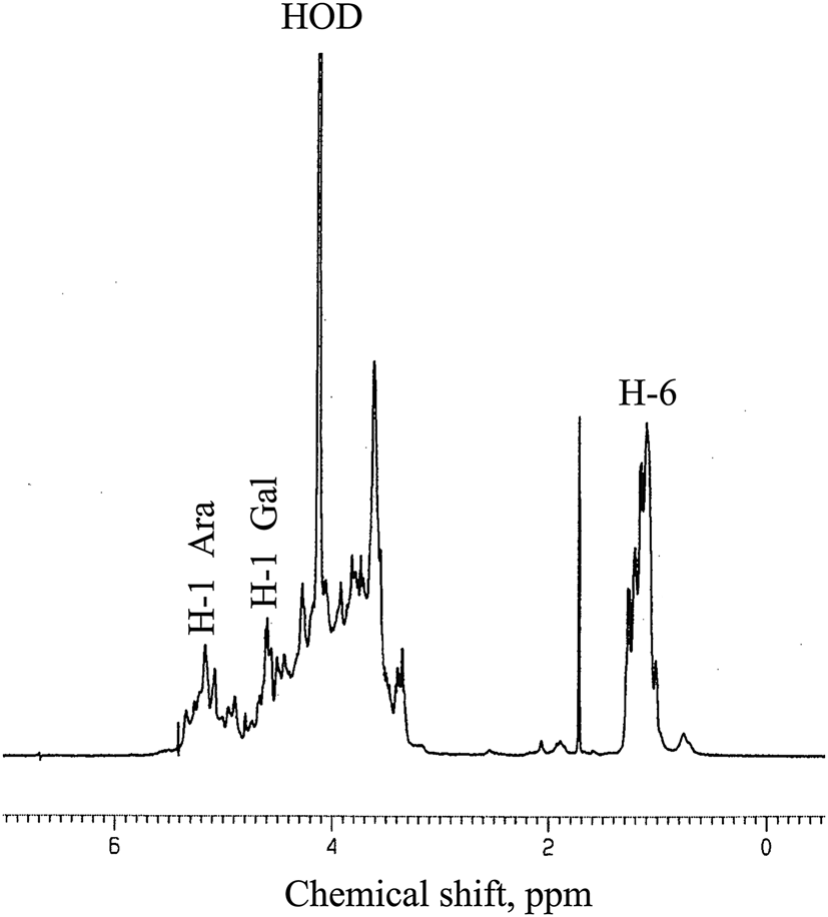
^1^H NMR spectrum at 600 MHz of the arabinogalactan (AF2) of *Cinnamomum zeylanicum.* The spectrum was recorded at 80 °C for sample in D_2_O solution. H-1α, H-1β, and H-6 refer to anomeric signals of α-linked, β-linked sugars, and methyl protons of arabinogalactan residues, respectively. The signal for the residual water was designated as HOD

The ^1^H NMR spectrum of the pectic arabinogalactan of present study is shown in Fig. 4. The presence of a large number of anomeric signals in the anomeric region suggests that the structure of this polymer is very complex. The signals appearing in the region between 4.98 and 5.26 ppm are resonances of the anomeric protons of different α-linked arabinofuranosyl residues (Habibi et al. 2005). The signals at *δ* 5.39 may be attributed to the resonance of H1 of 5-linked L-arabinofuranosyl residues (Nunes et al. 2008). A number of spin systems attributable to the anomeric proton of the *β*-galactopyranosyl residues appeared in the region *δ* 4.51–4.92 ppm. It also includes resonances of the ring protons that appear in the region *δ* 3.5–4.2 ppm. The high proportion of galacturonic acid residues must be responsible for some of the signals in the spectrum, but is not possible to assign any particular signals to these residues. It can be safely said that the ^1^H NMR spectrum of this polysaccharide is complex, overlapping, and inconclusive for structural information.

### Antioxidant activity of polysaccharides

#### FRAP assay

In recent years, many different methods are being used to evaluate antioxidant capacity of foods and biological samples (Huang et al. 2005). In some of these protocols, antioxidant assays were performed in alcoholic solutions, but in this condition polysaccharide would precipitate. Therefore, the antioxidant capacity of pectic polysaccharides (AF2, *B* and *C*) from *Cinnamomum zeylanicum* was determined by the FRAP assay. This assay in which a ferric salt Fe(III) (TPTZ)_2_Cl_3_ (TPTZ = 2,4,6-tripyridyl-s-triazine) is used as oxidant (Benzie and Strain 1996) takes advantage of electron transfer reactions (Huang et al. 2005). FRAP values increased considerably from 4 to 30 min, as it has been described for other vegetable and seaweed samples (Ruperez et al. 2002).

Regarding antioxidant capacity of the polysaccharides of present study, it is clear that the arabinogalactan (AF2) showed the highest reducing power at 4 and 30 min (155 and 180 μmol Fe(II) per gm sample dry weight, respectively). This is followed by the GalA-containing and glucan-containing fraction (Table 3). Results are expressed as μmol Fe(II)/g sample dry weight. For comparison of potencies, values are also calculated as μmol Trolox/g sample dry weight from regression equations as described by Pulido et al. (2000) of Trolox at 4 and 30 min of reaction with the FRAP reagent.

**Table 3:** Ferric ion reducing ability of soluble polysaccharide-containing fractions from *Cinnamomum zeylanicum*

Fraction	4 min		30 min	
	Trolox/g (μmol)	Fe(II)/g (μmol)	Fe(II)/g (μmol)	Trolox/g (μmol)
AF2^x^	155.3 ± 5.2^a^	89.6 ± 4.2^d^	180.1 ± 2.4^b^	86.3 ± 2.3^b^
*B* ^x^	92.1 ± 2.1^b^	53.3 ± 3.2^e^	137.1 ± 1.3^c^	85.1 ± 0.7^f^
*C* ^x^	78.0 ± 1.4^c^	32.3 ± 2.3^b^	88.8 ± 0.2^f^	42.1 ± 2.1^b^

Results are expressed as equivalent of Fe(II) or Trolox per gram dry weight of fractions in aqueous solution. Values represent mean of triplicates ± standard deviation. Superscript means with different letters are significant different to each other in the same column (*p* = 0.05)

^x^For a description of fractions obtained, see “Materials and Methods”

#### Scavenging effect on 1,1-diphenyl-2-picrylhydrazyl (DPPH) radicals

**Fig. 5:**
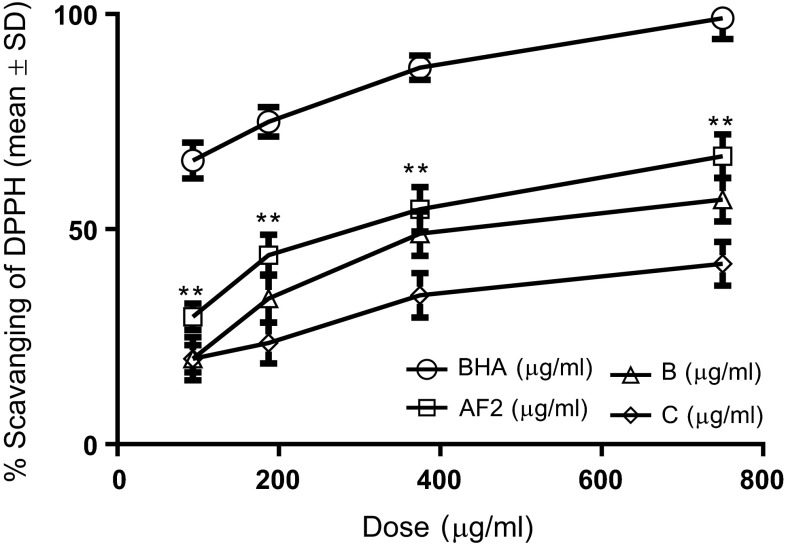
Scavenging effect of polysaccharides isolated from the barks of *Cinnamomum zeylanicum* on DPPH radicals. *BHA* butylated hydroxyanisole, *AF2* the arabinogalactan-rich fraction, *B* the galacturonic acid-containing fraction, *C* the glucan-rich fraction. Each value represents mean ± standard deviation (*n* = 3)

For further insight into the activation mechanism, we examined whether the protective effect was associated with DPPH radicals. The proton radical scavenging action is known to be one of the various mechanisms for antioxidation. DPPH is one of the compounds that possess a proton free radical and shows a characteristic absorption at 517 nm (purple) (Matsukawa et al. 1997). When DPPH encounters proton radical scavengers, its purple color would fade rapidly (Yamaguchi et al. 1998). An excellent scavenging capability on DPPH radicals at a dosage of 93.75 μg/mL (29.65 ± 3.14 %) [Each value represents mean ± standard deviation (*n* = 3)] was found with the arabinogalactan fraction AF2 as compared to the control BHA (65.98 ± 4.21 %) regarding the low dosage ranges used (Fig. 5). More significant and effective radical scavenging capability (66.98 ± 5.08 %) was also found with the fraction AF2 at a higher dosage (750 μg/mL). Comparable results with the fraction AF2 were found to be 52.87 ± 5.08 % at the dosages of 750 μg/mL for the GalA-containing fraction *B* (Fig. 5). By contrast, the glucan-containing fraction C demonstrated least scavenging capability 41.98 ± 5.08 % among three fractions at the concentration of 750 μg/mL. Moreover, the marked inhibitory effect of these polysaccharides on DPPH radicals was found to be concentration dependent (Fig. 5), although the activities were low. These results reveal that the polysaccharides of present study are potent scavenger and their antioxidative activity may be attributed to their proton-donating ability (Shimada et al. 1992). Probably, these fractions might contain a higher amount of reductone which could react with radicals to stabilize and terminate radical chain reaction. Whether the structures can be related to scavenging capacity for DPPH radicals remains to be determined in future studies. However, our preliminary results suggest that different bioactivities of these fractions apparently may be, in some respects, linked to their different molecular structures.

Most of earlier data on antioxidant of foods and biological samples were on phenolic compounds, and many researchers have been reported positive correlation between free radical scavenging activity and total phenolic compound. But this correlation between structure and activity is not always valid. For example, selected enzymatic extracts of seaweeds (*E. cava* and *S. coreanum*) did not possess antioxidant activity, although they contained as much phenolic compounds as the other extracts of *E. cava*. Feruloyl oligosaccharides showed a higher antioxidant capacity than free ferulic acid (Yuan et al. 2005). Moreover, the former showed greater antioxidant capacity in vivo than in vitro when compared to vitamin C (Ou et al. 2007). Therefore, it is believed that other materials in seaweed extracts, such as small molecular weight polysaccharides, pigments, proteins or peptides, may influence the activity. Indeed, recent data showed that a number of polysaccharide-containing fractions isolated from various sources such as higher plants (Aguirre et al. 2009), medicinal mushrooms (Jiang et al. 2008; Shimada et al. 1992), and even some enzymatic extracts (Je et al. 2009) possess antioxidant activity. Our results are in agreement with these studies. In addition, the polysaccharides used in this study were purified, and therefore, conclusively prove their antioxidant potency.

Although the antioxidant capacity of polysaccharide has been proved, the relationships between structure and antioxidative capacity have not yet been elucidated. This is primarily due to the interplay of two important factors. Firstly, the huge structural diversity of these polysaccharides has given a major hindrance in the structure–activity relationship establishment. However, on the basis of the accumulated data, several common structural motifs emerge that are important for activity. Secondly, many of the hitherto used pectic polysaccharides contained a number of other molecules. These later molecules may have their own activities and or at least dilute the efficacy of the sulfated polymer itself. However, the polysaccharides described in this work are purified and therefore conclusively prove their antioxidant activities.

## Conclusions

In this particular case, we found that the soluble polysaccharides of *Cinnamomum zeylanicum* can be an excellent antioxidant. The arabinogalactan exhibit more potency than the glucan, and therefore, this potency may be directly related with the molecular mass of these polysaccharides. These differences can as well be attributed to the differences in structure and perhaps to the actual availability of the functionality in each individual structure. The antioxidative activity of *Cinnamomum* polysaccharides may be attributed to their proton-donating activity as evidenced through DPPH radical scavenging results.

Because the polysaccharides tested in this study were basically prepared without toxic chemical reagents, it can be assumed to be potentially useful as a safe-antioxidant in food processing industries. Furthermore, as the isolation of these polysaccharides involves a few inexpensive and easy steps, it will be of an added advantage.

## Electronic supplementary material

Below is the link to the electronic supplementary material.

Supplementary material 1 (DOC 81 kb)
