# Supplementary material for: Antioxidant activity and structural features of Cinnamomum zeylanicum
Source: 3 Biotech. 2015 Mar 20;5(6):939–47. doi: 10.1007/s13205-015-0296-3 (PMC4624148; doi:10.1007/s13205-015-0296-3)
Supplement: Supplementary file 1 — Supplementary material 1 (DOC 81 kb) [file 13205_2015_296_MOESM1_ESM.doc]

**Supplementary Data for Reviewers**

**Supplementary Fig. 1.** GLC-MS analysis of the partially methylated alditol acetates (PMAA) derived from the arabinogalactan (AF2) of *Cinnamomum zeylanicum*. 1, T-Ara; 2, 1,2-Ara; 3, 1,2-Rha; 4, 1,3-Rha; 5, 1,5-Ara; 6, 1,2,4-Rha; 7, 1,3,5-Ara; 8, 1,3-Gal; 9, 1,4-Glc; 10, 1,6-Gal; 11, 1,3,6-Gal.

5

7

8

9

10

11

4

2

1

6

3

12

3

1

4

5

10

11

2

3

7
